# Charting new courses to enter foreign markets: Conceptualization, theoretical framework, and research directions on non-traditional entry modes

**DOI:** 10.1057/s41267-022-00521-x

**Published:** 2022-04-22

**Authors:** Keith D. Brouthers, Liang Chen, Sali Li, Noman Shaheer

**Affiliations:** 1 King’s Business School, King’s College London, London, UK; 2 Faculty of Business and Economics, The University of Melbourne, Melbourne, Australia; 3 Darla Moore School of Business, University of South Carolina, Columbia, USA; 4 Discipline of International Business, University of Sydney, Sydney, Australia

**Keywords:** entry mode, digital, platform ecosystems, embeddedness, networks, capital market

## Abstract

Recent advances in digitalization and increasing integration of international markets are paving the way for a new generation of firms to use non-traditional entry modes that are largely marginalized in previous entry mode studies. While extant research revolves around the level of resource commitment and control in foreign activities, non-traditional modes are encapsulated by the extent of embeddedness required for exploring new and/or exploiting existing resources. In particular, we draw attention to four such categories of non-traditional entry modes the literature has touched on, i.e., capital access, innovation outposts, virtual presence, and the managed ecosystem. We explore the key attributes, antecedents, and strategic implications of these modes. Our paper highlights the need for enriching current entry mode research by considering a broader range of entry mode activities available to firms as well as employing new theoretical perspectives to understand the complex phenomena of internationalization.

## INTRODUCTION

International entry or operation mode has been one of the defining research topics for international business (IB), with numerous classic articles claiming the Decade Award at the *Journal of International Business Studies* (e.g., Agarwal & Ramaswami, 1992; Anderson & Gatignon, 1986; Brouthers, 2002; Kogut & Singh, 1988). A notable review of this literature suggests that international entry modes are commonly characterized as governance structures by which the MNE organizes its value-adding activity in foreign markets (Brouthers & Hennart, 2007). Since the early work of Anderson and Gatignon (1986), Hennart (1982), and Williamson (1985), a number of theoretical perspectives have been used to explain entry mode choice (e.g., Brouthers, Brouthers, & Werner, 2008; Meyer, Wright, et al., 2009). Nonetheless, the literature continues to be dominated by transaction cost theory (TCT), which maintains that the alignment between different governance structures and transactional characteristics determines a firm’s entry mode choices (Cuypers, Hennart, Silverman, & Ertug, 2021; Zapkau, Schwens, & Brouthers, 2018).

While TCT has much advanced our understanding of foreign entry from the ‘firm boundary’ perspective, it only predicts that *given* firms’ strategic goals, they will choose the arrangement that economizes on transaction costs. A wide adoption of TCT unnecessarily restricts the conceptualization of entry modes to categories of organizational structures that serve to mitigate contractual concerns associated with specific cross-border transactions (Pan & Tse, 2000). The conceptual lens has also resulted in the majority of empirical entry mode studies being confined to the choice between shared and full ownership modes (Brouthers & Hennart, 2007; Canabal & White, 2008; Zhao, Luo, & Suh, 2004). Despite the variety of modes of entry that were historically examined by IB researchers (including several JIBS Decade Award articles such as Bilkey, 1978; Davidson & McFetridge, 1985; Reid, 1981), many of these modes have received, in the past few decades, far less attention in mainstream entry mode studies, both theoretically and empirically (Brouthers & Hennart, 2007; Canabal & White, 2008). As reported by a recent review, limited progress has been made in expanding the scope of entry mode scholarship (Zapkau et al., 2018), making researchers question whether this literature is indeed getting ‘more of the same’ (Shaver, 2013).

Nevertheless, there are two recent, distinct trends indicating where extensions of the entry mode literature are holding most promise. First, the emergence of such modern phenomena as digital technologies, changes in global and national institutional frameworks and the development of global value chains has drawn significant attention to forms of international economic *involvement*, as “the scale and scope of the modern firm becomes a question of involvement and not only of investment” (Liesch, Buckley, Simonin, & Knight, 2012: 15). This has bred a renewed interest in non-traditional international entries that no longer represent structures for reducing cost and instituting administrative control (Teece, 2014). For instance, digital platform ecosystems create a new channel for traditional firms to enter international markets (Chen, Li, Wei, & Yang, 2022a; Deng, Liesch, & Wang, 2021; Jean, Kim, Zhou, & Cavusgil, 2021; Nambisan, Zahra, & Luo, 2019). The deregulation of the financial industry has also better enabled firms to access capital markets in foreign countries. Second, the literature has shown a growing proliferation of theoretical perspectives, with comparatively more emphasis on the resource-based view (RBV) and institutional theory (Brouthers, et al., 2008; Zapkau et al., 2018). For instance, drawing on the RBV, Meyer, Wright, et al. (2009) re-classify international entry modes by the degree of resource exploitation vs resource augmentation (a form of exploration) in foreign markets, instead of the firm’s boundary decision as motivated by transaction cost minimization. These two emerging trends suggest that, as Liesch et al., (2012: 15) submit, we need “a complementary explanation to internalization theory” to account for those entries involving limited local asset ownership.

In this paper, we take stock of recent developments in the literature on international entry to highlight a growing trend in the use of non-traditional entry modes. We seek to incorporate the modern theory of MNEs that define foreign entries and operations as network-based approaches to deploying existing capabilities or accessing new capabilities (Teece, 2014). Following this view, we identify four prominent mode categories: capital access, innovation outpost, virtual presence, and managed ecosystem entry modes, each of which helps us classify an array of entry modes possessing unique attributes that we believe can be better explained through the application of theories focusing on (a) deployment of existing capabilities or access to new capabilities (Teece, 2014) and (b) firm ‘involvement’ instead of investment into foreign markets (Liesch et al., 2012).

We contribute to the literature on three fronts. First, we present the state-of-the-art of the entry mode literature and reveal a growing trend in recent research that sheds new light on non-traditional modes of international entry, which do not solely serve as a vehicle for transaction cost minimization. Second, we highlight key differences between the non-traditional mode categories we identify and those traditional ones. In considering their relationships, we elucidate how the renewed focus on non-traditional entries can revive our conceptual development beyond the received theoretical lenses. Third, we build on ideas put forth by leading scholars (Liesch et al, 2012; Teece, 2014) to introduce a new framework that synthesizes emerging research on non-traditional entry modes deployed by modern internationalizing firms. We show how our framework can inspire future research.

## ENTRY MODE RESEARCH

Transaction cost theory continues to shape much of the received wisdom around internationalization and suggests that the *raison d’etre* of the MNE is to achieve efficiency from the internal transfer of technology and intermediate goods when factors of production and technology are given (Casson, 1986; Hennart, 1982; Narula, Asmussen, Chi, & Kundu, 2019). As a result, traditional entry mode research has long framed entry modes as organizational structures for mitigating contractual concerns in the exploitation of firm-specific advantages and to a lesser extent for the exploration of new resources and knowledge (Casson, 1986; Hennart, 1988, 1989). This is despite the fact that by definition entry modes should include all arrangements of firm participation, or ‘involvement’, in a foreign market (Brouthers & Hennart, 2007).

Following this traditional view entry mode considerations are based predominantly on control, commitment, and risk (Tse, Pan, & Au, 1997; Zhao et al., 2004), to the extent that researchers often refer to entry modes as “control strategies ranging from full ownership to market relationships (that) are used to coordinate global activities” (Buckley & Ghauri, 2004: 82). According to TCT, asset specificity, uncertainty and appropriability affect the cost of arm’s length transactions and determine the quest for managerial control (Cuypers et al., 2021). For instance, joint ventures are deemed a type of internalization by which firms can “obtain effective control with less than full ownership” (Hennart, 1991b: 491). In this regard, the definition of the MNE as “a firm that owns and controls activities in two or more different countries”, links entry modes to the very core of IB (Buckley & Casson, 2009: 1564).

While the entry mode literature represents one of the most established areas of research in IB, our conceptual understanding of modes continues to evolve and remains incomplete for several reasons. First, notwithstanding the dominance of received theories like TCT and RBV, researchers have revealed an increasing proliferation of theoretical perspectives (Martin, 2013; Zapkau et al., 2018), such as institutional theory (Davis, Desai, & Francis, 2000; Lu, 2002; Yiu & Makino, 2002) and network theory (Filatotchev, Strange, Piesse, & Lien, 2007), suggesting that new insights can be gained by moving beyond conventional lenses of cost minimization and asset exploitation (Kim & Hwang, 1992). For example, Elia, Larsen, and Piscitello (2019) employ a behavioral economics perspective (e.g., heuristics and biases) in explaining why and when entry mode decisions may deviate from transaction cost predictions, a common phenomenon identified by earlier research (e.g., Brouthers, 2002). Others draw on a capabilities view to associate mode choices with firm-specific strategic considerations such as access to new capabilities (Chen, 2008; Chen & Hennart, 2002) and management of knowledge flows (Contractor & Kundu, 1998; Martin & Salomon, 2003). Nevertheless, TCT continues to dominate our thinking to the extent that foreign subsidiaries are often viewed as the preferred organizational structure because of their capacity to protect from misappropriation firm advantages to be exploited (Hennart, 1991a), while much less attention is paid to modes of entry in regard to capability augmentation (Cantwell & Mudambi, 2005; Frost, Birkinshaw, & Ensign, 2002).

Second, there is notable disagreement in the literature on how to categorize entry modes (Hennart & Slangen, 2015). Prior literature reviews report that studies of cooperative modes often conflate equity-based and contractual cooperation and instead focus on comparing full control with shared control modes (Canabal & White, 2008; Morschett, Schramm-Klein, & Swoboda, 2010). While most empirical studies examine firms’ selection decisions between two or more alternative modes (e.g., equity vs non-equity modes), those archetypal entry modes are in fact *categories of modes* that researchers assume belong in the same choice set for a firm (Martin, 2013; Pan & Tse, 2000). For instance, joint ventures, one of the most widely studied mode choices, represent a distinct category incorporating various types of establishments (e.g., majority, equal and minority owned) in which the focal MNE will face different challenges (Blodgett, 1991; Cuypers & Martin, 2009; Franko, 1989). Other traditional entry mode categories include exporting (Li, He, & Sousa, 2017), licensing (Arora & Fosfuri, 2000; Mottner & Johnson, 2000; Shane, 1994), franchising (Brickley & Dark, 1987; Fladmoe-Lindquist & Jacque, 1995; Rosado-Serrano, Paul, & Dikova, 2018), and contracting and outsourcing (Contractor & Kundu, 1998; Erramilli & Rao, 1993). Aulakh and colleagues, for instance, investigate differences within the category of international licensing agreements such as exclusivity rights, licensing duration, and compensation structures (Aulakh, Cavusgil, & Sarkar, 1998; Aulakh, Jiang, & Li, 2013; Jiang, Aulakh, & Pan, 2009). Although the FDI portion of entry mode studies frames ownership as a means of containing transaction costs (Brouthers, 2013), much of the non-equity mode literature tends to focus on efficient transfer of capabilities instead of control of operations as the main driver of mode choices (Erramilli, Agarwal, & Dev, 2002). Within these various traditional entry mode categories, firms need to make selections between alternative institutional arrangements that are characterized by, *inter alia*, varying levels of equity ownership, financial commitment, control, and risk.

Third, historically IB research has shown a non-trivial interest in various ‘exotic’ modes of ‘involvement’, or so-called ‘new forms of entry’, that substituted for traditional FDI (Hennart, 1989). For example, free-standing firms, which refer to those operating major business activities in one country but raising equity funds in another, have been explored by a few scholars (Casson, 1994; Hennart, 1994; Wilkins & Schroter, 1998), but have not been widely discussed in the entry mode literature. Yet recent trends indicate that accessing foreign financing without further entry has become a popular way for many firms to gain the resources they need for business growth (Purkayastha & Kumar, 2021). Another example of these non-traditional entry modes is the literature looking at the impact of the internet on foreign entry (Zaheer & Manrakhan, 2001). These studies suggest that firms can take advantage of the internet as a lower cost mode of entry (Nachum & Zaheer, 2005). Furthermore, as the rate of change in technology has continued to accelerate, firms have begun using ‘outposts’ as an entry mode to gain knowledge about potential technology changes that facilitate modification of firm-specific assets or products to enhance advantages (Chung & Alcácer, 2002; Mudambi, 2008). Yet again these new forms of foreign country involvement have seen little discussion in the traditional entry mode literature leading Hennart and Slangen (2015) to conclude, our knowledge of entry modes remains incomplete.

That said, changes in the international business landscape over recent decades have potentially revitalized the use of these and other similar non-traditional modes by which MNEs participate in foreign markets. For instance, digitalization (not just the internet) of product offerings and business processes has drawn scholars’ attention to new ways of non-equity governance (Lew, Sinkovics, Yamin, & Khan, 2016) and to new approaches of servicing foreign markets from a distance (Autio, Mudambi, & Yoo, 2021). The emergence of global value chains has also bred a growing interest in outsourcing and R&D alliances (Kano, Tsang, & Yeung, 2020), and raised the importance of the firm’s role in searching for new capabilities and of inward flows of knowledge. Although Teece (2014) claims that such a strategic motivation is beyond the realm of entry mode research, the broader IB scholarship does examine firms’ embeddedness in foreign business networks and how it affects their ability to develop new competences (Andersson, Forsgren, & Holm, 2002; Vahlne & Johanson, 2017). Following this view, entry may be driven by access to new capabilities, and by implication, modes of entry will depend on network partners who own and control such capabilities (Figueiredo, 2011; Forsgren, 2016). Notably, in proposing a new classification of entry modes that departs from the transaction cost approach, Meyer, Wright, et al. (2009) delineate the capacity of various modes in enabling exploration of new knowledge vs exploitation of existing knowledge. Entry modes are thus conceptualized as vehicles for transferring (and sourcing) knowledge, instead of a governance structure for the exchange of intermediate goods or complementary assets in a specific transaction.

**Figure 1:**
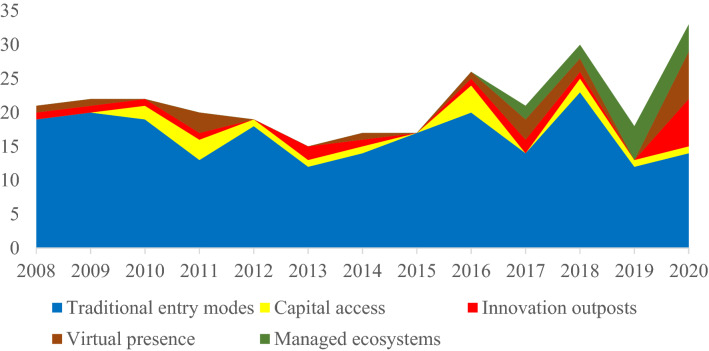
Number of articles published each year on traditional and non-traditional entry modes in major IB and management journals.

These recent transitions in entry mode choices available to MNEs have started attracting substantial research attention with several studies exploring non-traditional modes of firm engagements in foreign markets. This emerging literature, however, is rarely integrated or even recognized in mainstream entry mode research. In particular, we draw attention to four prominent categories in the current literature – capital access, innovation outposts, virtual presence and managed ecosystem, each pertaining to a distinct non-traditional mode of foreign entry. Based on an extensive survey of the major IB and management journals (post Brouthers & Hennart, 2007), we plot in Figure 1 the number of articles published each year on traditional entry modes and show the growing interest in each category of non-traditional entry mode.

Below, we synthesize research under each non-traditional entry mode category to highlight the antecedents and key attributes of these modes. Next, we seek to reinvigorate entry mode scholarship by providing a new theoretical framework for synthesizing these non-traditional modes. Finally, we offer some directions for future research that would contribute to our knowledge about how modern businesses leverage new technologies and global realities to enter foreign markets. We expect that synthesizing the nascent but fast emerging inquiry of non-traditional entry modes and integrating it with mainstream entry mode research will open broader avenues for future research and help cross-fertilize with other disciplines, such as information systems and marketing, in understanding modern ways of foreign operations.

### Capital Access

Capital access entry modes refer to foreign market entries for seeking and gaining access to new financial resources while at the same time undertaking little, if any, other activities in the foreign market. We term these non-traditional entry modes ‘capital access’ modes as the key purpose of such market entries is pursuit of financial resources. Indeed, capital access mode reverses the traditional conceptualizing of foreign entry; instead of investing into a foreign country, firms receive foreign investments for conducting business in domestic and/or other international markets.

There are a large variety of means through which firms can access foreign capital such as initial public offerings (IPO), seasoned equity offerings (SEO), cross-listings in foreign stock markets, bank loans, foreign bond issues, engagement with private equity firms, international investment syndicates, foreign venture capital (VCs), sovereign wealth funds (SWFs), as well as several informal equity capital channels (Filatotchev, Bell, & Rasheed, 2016). While traditional entry mode research focuses on firm entries into foreign markets to sell products or services or to gain access to location-specific knowledge/resources, the easing of financial flows across borders is enabling firms to seek capital resources from other countries even without entering these product/services markets (Bell, Filatotchev, & Rasheed, 2012). Though it is not a completely novel mode of entry and has been studied by different streams of research, recent transitions in financial regulations, emergence of international venture capital firms, and ease of financial flows across countries have made this task easier and more common. For example, companies such as Alibaba and Aramco sought IPOs in the US without operating or having physical assets in the “host” country (Franklin, 2020). Similarly, many start-ups and other young ventures receive funding from foreign VCs while staying in their home countries (Humphery-Jenner & Suchard, 2013; Liu & Maula, 2015) and these firms are more likely to subsequently list on foreign exchanges. As a result, we observe a recent surge in research interest on such entries.

Studies examining the antecedents to capital access entry mode suggest that firms are more likely to seek and obtain foreign capital if their home countries encourage innovation, guarantee regulatory stability, have advanced product markets, have well-functioning labor or capital markets, and/or protect investor rights (Guler & Guillén, 2010b; Pezeshkan, Smith, Fainshmidt, & Nair, 2020). In contrast, some research found that it is more difficult for firms from emerging countries or countries with low quality institutions to undertake capital access mode of entry (Buchner, Espenlaub, Khurshed, & Mohamed, 2018; Mingo, Morales, & Dau, 2018). Nevertheless, firm-level antecedents are equally important as firms pursuing capital access entry mode typically have high growth expectation, high R&D expenditures, patents, and are from smaller countries (Schertler & Tykvová, 2011). Firms also try to access foreign capital through syndicates where foreign investors co-invest with local investors, particularly when the distance between firm’s home country and foreign investors is high and foreign investors can find suitable domestic co-investors (Dai & Nahata, 2016; Khavul & Deeds, 2016; Luo, Rong, Yang, Guo, & Zou, 2019; Tykvová & Schertler, 2014).

Researchers have also investigated the characteristics of foreign investors with whom firms may engage to undertake capital access mode. Studies show that firms are more likely to attract investors having more extensive international experience and relationships with international investment syndicates (De Prijcker, Manigart, Wright, & De Maeseneire, 2012; Schertler & Tykvová, 2011) or investors who enjoy high social status in their home-country networks or have network partners operating in foreign countries (Guler & Guillén, 2010a) .

While the capital infusion provided by foreign countries might take the dominant role, capital access mode also helps firms obtain strategic advantages from overseas investors and lenders such as knowledge and managerial assistance, improved corporate governance, and positive reputation (Pagano, Röell, & Zechner, 2002; Stulz, 1999; Tykvová & Schertler, 2014). Several studies have evaluated the consequences for entering foreign markets to access financial resources and the related knowledge benefits. Cumming, Knill, and Syvrud (2016) report that successfully obtaining foreign capital leads to higher IPO proceeds in the future as foreign investors contribute new knowledge and expertise. However, the potential knowledge benefits from accessing foreign capital are not without challenges. Dai and Nahata (2016) and Gu and Lu (2011) point to the difficulty of taking advantage of the expertise of foreign investors when cultural distance is high but suggest this can be overcome when the investors have extensive experience in domestic markets or when firms receive co-investments from both foreign and domestic investors.

### Innovation Outposts

Innovation outposts entry modes represent foreign entries for exploring resources and capabilities in foreign countries by setting up a listening post and earning an insider status in networks of foreign firms. Innovation outposts refer to dedicated teams managing inflows and outflows of knowledge that foster innovation for the firm (Decreton, Monteiro, Frangos, & Friedman, 2021). Firms undertaking this mode establish foreign innovation outposts, which allow firms to explore for new innovative ideas about technologies, business models and management knowhow that are unavailable internally or in the business networks they are already exposed to.

Innovation outpost entry mode is related to capital access entry mode as innovation outposts also allow firms to seek and access new resources and capabilities in foreign markets, yet they differ from capital access entry modes in two important ways. First, the focal firm must establish some operations in the foreign country to make the entry mode work, though these operations do not require foreign direct investments, establishment of foreign subsidiaries or sales office, or even formal contractual arrangements. Second, firms entering via innovation outposts do not seek financial resources but are interested in knowledge about new technology, innovation in products and processes, and managerial competencies. To some extent, innovation outposts entry modes can also be considered an extension of international alliances research. Alliance research has traditionally focused on foreign market-specific knowledge that firms seek from foreign supplier and distributor networks cultivated through traditional entry modes such as exports, outsourcing, alliances and joint ventures (Chetty, Eriksson, & Lindbergh, 2006; Johanson & Vahlne, 2009). Nevertheless, scholars increasingly recognize deeper nuances of firms’ network embeddedness, implying that firms may seek knowledge and resources that are not necessarily market-specific and can be deployed across multiple countries to improve products, services, and business processes (e.g., Cantwell, 2009; Cuypers, Ertug, Cantwell, Zaheer, & Kilduff, 2020; Kostova, Marano, & Tallman, 2016; Vahlne & Johanson, 2020). Searching for and accessing such knowledge and resources may not necessarily require traditional arrangements like subsidiaries, sales offices or formal contractual relationships with suppliers or distributors. Instead, firms may get access to such networks by locating internal teams in foreign countries, (i.e. innovation outposts), which may cultivate relationships with foreign business networks to access their unique knowledge and resources (Decreton et al., 2021). Unlike signing a one-time contract for alliances or joint ventures, building these relationships is a continuous process that gradually enhances firm embeddedness in a foreign network through reciprocal and informal commitments (Chandra & Wilkinson, 2017; Forsgren, 2016; Johanson & Vahlne, 2011; Santangelo & Meyer, 2017; Vahlne & Johanson, 2017). Innovation outposts entry modes are particularly critical in a digital age where the unprecedented connectivity is enhancing firm capacity to orchestrate partner networks without traditional entry (Coviello, 2006; Verbeke & Hutzschenreuter, 2020; Watson, Weaven, Perkins, Sardana, & Palmatier, 2017; Zaheer & Manrakhan, 2001).

Given the importance of network embeddedness for innovation outposts entry modes, research most relevant to these entry modes discusses firms searching for and accessing managerial and technical resources in foreign countries by overcoming their liabilities of outsidership (LOO) to embed in foreign business networks (Coviello & Munro, 1997; Forsgren, 2016; Vahlne & Johanson, 2020). We note the critical need for extending this research stream to better understand innovation outpost entry modes. Below, we extract relevant insights from the literature, which may guide research on these entry modes.

First, we draw attention to research pertaining to the benefits firms may seek through innovation outposts entry modes. Dong, Li, and Tse (2013) show that a combination of formal governance mechanisms and informal relationships with foreign partners is more likely to help foreign firms take advantage of partner capabilities and networks. Similarly, Khavul, Peterson, Mullens, and Rasheed (2010) suggest that strategically cultivating foreign networks through extensive interactions and information exchanges may help firms explore new ideas.

To achieve these benefits from innovation outposts entry modes, firms need to establish an insider position in business networks of a target country. Such networks are based on mutual interests and ongoing relationships that differ from the explicit contracts in alliances and joint ventures or establishment of subsidiaries, reflecting the transition from earlier worldwide hierarchies to devolved, network-like organizational systems in which not only top management but also middle management is heavily involved (Chandra & Wilkinson, 2017; Sandberg, 2014). Hence, firms move beyond foreign investments and hierarchal controlled subsidiaries to develop networks with external firms in foreign countries, which help them explore knowledge and resources in foreign countries. These relationships and implications of knowledge and resources gained through them can transcend national borders. Cantwell (2009) reveals the complex nature of integrated and interactive networks firms cultivate across different locations in order to combine specialized knowledge and resources from multiple countries for generating novel strategic advantages. Cuypers et al. (2020) discuss the possibility of a born-global phenomenon where firms may leverage technology to simultaneously enter relevant networks across multiple foreign countries. Similarly, Connelly, Ketchen, and Hult (2013) highlight that firms can integrate knowledge gathered from networks across different countries to develop novel products, practices, and business models. Hult, Gonzalez-Perez, and Lagerström (2020) advocate expanding the notion of networks to include international business ecosystems, defined as stakeholders, organizations, and countries involved in exchanges, production, business functions, and cross-border trade through both marketplace competition and cooperation.

The main challenge behind innovation outposts entry mode is to gain an insider status in foreign business networks by overcoming LOO. Recent research subscribes to the view that overcoming LOO to enter a foreign network may not be a discrete event like a traditional entry but a continuous process that requires a longitudinal and dynamic perspective (Santangelo & Meyer, 2017; Vahlne & Johanson, 2017). Kurt and Kurt (2020) suggest that overcoming LOO requires firms to pay closer attention to the structural characteristics of foreign business networks in which it wants to enter; insidership in closed networks requires more resources and time than open networks but the tacit and sensitive knowledge from trust-based closed networks may be vital when distance is high. In a similar vein, Forsgren (2016) argues that existing networks may help firms overcome LOO in new networks across countries, though extensive embeddedness in current networks may also leave firms with lower resources for overcoming LOO in additional countries. Li and Fleury (2020) focus on the importance of absorptive capacity for overcoming LOO. They suggest that the selection of partners with the proper level of knowledge overlap, as well as engagement with users and local communities, is critical for gathering required knowledge to overcome LOO and benefit from foreign networks. Stoyanov, Woodward, and Stoyanova (2016) draw attention to diaspora networks in foreign countries, which may help foreign firms overcome their LOO.

### Virtual Presence

Virtual presence entry mode refers to foreign entries in which no physical entry is undertaken for customer/user acquisition, although the firm appears to be in the country from the perspective of its buyers and users. This mode of entry highlights how firms can leverage existing advantages in foreign markets while maintaining little or no physical presence in the country. Virtual presence entry mode is made possible due to recent advances in digital technologies that enable firms to enter foreign countries by directly acquiring customers or users and often delivering products (3D printing) or services (downloads) while actually avoiding the need to establish formal foreign-based subsidiary units or export channels (Monaghan, Tippmann, & Coviello, 2020; Shaheer & Li, 2020). Direct access to customers/users is particularly prominent for digital service firms who, without any local establishments, can exploit their digital technologies in any country by acquiring customers/users through digital channels. Even product-based firms can use digital technologies to enter foreign markets with no foreign presence either by establishing a single-sided platform or by becoming a complementor to a multi-sided platform firm that has already entered the foreign market (see managed ecosystem entry mode). Firms may disseminate physical products to customers/users without being locally present through digital technology or using international delivery services (Coviello, Kano, & Liesch, 2017; Laplume, Petersen, & Pearce, 2016). Payment services like PayPal and Alipay further reduce the need of local operations as they facilitate monetary exchanges in any currencies on global platforms (Li, Chen, Yi, Mao, & Liao, 2019). Based on these advances, a growing number of scholars stress the need to re-conceptualize foreign entry as a process of customer/user acquisition instead of investments or asset acquisition (Brouthers, Geisser, & Rothlauf, 2016; Chen, Shaheer, Yi, & Li, 2019; Shaheer & Li, 2020).

Traditional entry mode research has looked at how management consultancies, engineering services, or advertising agencies, can export their services by leveraging the emergence of global standards (Cheung & Leung, 2007; Dou, Li, Zhou, & Su, 2010; Jandhyala, 2013). Studies in this area have noted that for certain types of services (where production and use of the service can be separated), exporting from the home country provides a means of expanding abroad. Virtual presence entry modes differ from traditional modes like exporting, licensing or franchising in two ways. First, virtual presence entry modes do not require the firm to set up operations of any kind (including cooperative modes like export agents, distributors, or export sales subsidiaries) in the foreign market, instead relying on digital technologies to reach customers/users. Second, through these digital channels, firms can leverage their advantages reaching large groups of potential customers/users and retain all the financial benefits of foreign entry (instead of sharing with others), because virtual presence entry modes provide firms with the ability to enter foreign markets and directly control the operation while minimizing investment in the foreign market.

Research related to virtual presence entry modes focuses on foreign customer/user acquisition via digital channels. Pezderka and Sinkovics (2011) recognize foreign entries via digital channels as a distinct entry mode, arguing that firms may prefer exploiting their technologies via virtual presence entry modes instead of adopting traditional entry modes when they lack adequate resources and perceive higher risk. Cahen and Borini (2020) suggest that firms need certain expertise for virtual presence entry modes, particularly cross-cultural programming skills (i.e., development of interfaces that could adjust to different international markets without re-writing the codes every new market), global virtual networks, cross-border digital monetizing adaptability (i.e., adjusting revenue models to the local requirements in foreign markets) and international business model reconfiguration. However, they suggest that firms, after successful virtual entry, tend to subsequently strengthen their presence though more traditional, non-equity entries such as corporate offices or data centers. Jean and Kim (2020) emphasize the importance of firms’ internet capabilities for virtual presence entry modes.

Researchers also focus on host country factors that stimulate firms’ preference for virtual presence entry modes. Schu and Morschett (2017) show that firms prefer countries with attractive market size, low distance, and high institutional quality. Similarly, Schu, Morschett, and Swoboda (2016) highlight distance, risk of imitability, VC investment in a firm, and prior international experience as important factors. Jean, Kim, and Cavusgil (2020) find that firms may prefer virtual presence entry mode by participating in an online platform as a complementor when the risk of imitability, market volatility and competition in a foreign country is low.

Some scholars direct attention to the global availability of digital technologies and minimal cost of foreign market entry, characterizing virtual presence entry mode as a diffusion-based process in which user adoption of globally available digital technologies, instead of firm decisions, determines firm entries in foreign countries (Autio, 2017; Coviello et al., 2017; Shaheer, Li, & Priem, 2020). Shaheer and Li (2020) argue that foreign entry via digital channels is not without its challenges. They show that users may be reluctant to adopt technologies developed by foreign digital firms but engaging foreign users in co-creation may help firms enter a foreign country. Similarly, Monaghan et al. (2020) contend that digital firms can leverage increased automation to directly engage with foreign customers/users and other stakeholders, which may facilitate their entries in target countries. Reuber and Fischer (2009, 2011) stress the importance of firm-specific factors, particularly technological capabilities and reputation, in stimulating customer/user adoption in a new market. Shaheer et al. (2020) highlight the importance of lead markets that may help digital firms refine their technologies to be more appealing to customers/users in multiple countries. They suggest that digital firms may strategically enter lead markets first to facilitate subsequent foreign entries. Zhang, Song, and Qu (2011) indicate that the lower requirement of physical presence may not reduce competition as foreign entry requires digital firms to differentiate their technologies from local competitors and actively respond to competitive actions. In a similar vein, researchers also suggest that digital firms need to adjust their technologies to varying customer/user requirements across countries, based on national cultures and customer/user motivations behind technology usage, in order to create customer/user value and earn customer/user trust (Ashraf, Thongpapanl, & Auh, 2014; Nam & Kannan, 2020; Thongpapanl, Ashraf, Lapa, & Venkatesh, 2018).

### Managed Ecosystems

Managed ecosystem entry modes deal with foreign entries of multi-sided platforms, in which platform firms leverage their firm-specific digital infrastructure while also cultivating localized operations. Altman, Nagle, and Tushman (2021) define managed ecosystems as unique ways in which firms engage external parties for value creation and capture such that the locus of activity resides outside the focal firm’s organizational boundaries while the locus of control remains within the firm. Managed ecosystem entry modes differ from virtual presence entry modes although both provide a vehicle for the firm to leverage firm-specific advantages in a foreign market. Managed ecosystem entry modes, however, require the firm to establish a presence in the foreign country to comply with local legislation, gain legitimacy, attract local complementors, and create value for customers/users.

We identify substantive differences between managed ecosystem entry modes and more traditional modes as multi-sided platform firms can exploit firm-specific digital infrastructure as they create value by combining firm-specific advantages with complementary resources provided by local complementors in foreign countries (Brouthers et al., 2016). Reliance on complementors is different from traditional alliances or joint ventures as complementors are not hierarchically integrated partners or employees. Instead, they are independent contributors satisfying country-specific user needs and enhancing platform value in local markets, even without a direct financial relationship with the platform firm (Chen, Li, et al., 2022; Li et al., 2019; Vahlne & Johanson, 2017). We recognize substantial variations in the extent to which multi-sided platforms extensively rely on local complementors to create value (e.g., Just Eat, Airbnb, Uber, Groupon) or combine a mix of local and international complementors (e.g., TikTok, YouTube, App Store, etc.). Nevertheless, the value generated by these firms continuously evolve due to not only the design and configuration of the digital infrastructure provided by the focal platform firm, but also the contributions of complementors (Chen, Li, et al., 2022; Dattée, Alexy, & Autio, 2018; Nambisan et al., 2019). Such a combination of globally accessible digital infrastructures and network of independent complementors provides digital platform firms an entry mode for exploiting their firm-specific advantages in foreign markets.

There is a nascent but fast-growing research stream examining managed ecosystem entry modes. Primarily, scholars focus on the unique aspects of ecosystem platform-based entries. Nambisan et al. (2019) focus on two distinct platform features, the ability to connect internationally diverse complementors through a common digital interface and the flexibility to consolidate complementor contributions for adapting platforms to preferences in several countries. Such connectivity and flexibility enable platforms to leverage their complementor networks for addressing country-specific needs despite the lack of firm investment or first-hand experience in foreign markets. Similarly, Parente, Geleilate, and Rong (2018) argue that entry in a new country is used for exploiting firm-specific digital infrastructure and governance policies but that these firms leverage complementors in host countries to satisfy local needs. A prominent case of leveraging complementors to meet local needs is presented by Tran, Yonatany, and Mahnke (2016), who study Facebook developing a network of complementors across countries for crowdsourced translation of platforms in local languages.

Recognizing the importance of bundling firm-specific digital infrastructure with complementor networks across countries, Banalieva and Dhanaraj (2019) argue that ecosystem platform entry to new countries involves a governance choice about openness of digital platforms to complementor networks (Chen, Yi, et al., 2022), which is not only influenced by the nature of firm-specific advantages but also network-based advantages. Platform firms may enter foreign countries by centrally controlling their core technologies and activities requiring advanced human capital, while opening access to peripheral technologies to take advantage of the capabilities of their complementors and delegating activities requiring generic human capital to network partners. Li et al. (2019) highlight the role of ecosystem-specific advantages for managed ecosystem entry modes; platforms need digital infrastructure and effective governance to orchestrate a coherent ecosystem of loosely coupled firms and complementors, who provide complementary resources and positive externalities. Entering a country requires a platform to not only develop a new ecosystem in the host country but also enact a governance system to ensure a level of complementarity between the new ecosystem and already established ecosystems in other countries (Chen, Tong, et al., 2022).

Other research deals with challenges of developing and maintain managed ecosystem entry modes in foreign countries. For managed ecosystem entry modes, platforms need an initial set of complementors who can improve the local appeal of the platform in each country to attract more customers/users and complementors through positive network externalities. As Brouthers et al. (2016) indicate, social and trading platforms, termed as ibusinesses, face LOO due to their lack of presence in customer/user and complementor networks of foreign countries. They suggest that firms can overcome this LOO by cultivating large customer/user networks across multiple other countries as the content produced by diverse customers/users across countries may also meet customer/user preferences in a new country and help these platform firms acquire an initial set of customers/users and complementors. Subsequent research by Chen et al. (2019) shows that simply acquiring customers/users across multiple countries may not always help platforms overcome the LOO. Instead, these firms should establish a presence in high clout countries first which then facilitates the expansion of the platform to other countries by attracting local complementors and customers/users, subsequently leading to platform entry in new countries. In a similar vein, Ojala, Evers, and Rialp (2018) argue that regardless of cross-national distance or familiarity of platform firms with a foreign market, platforms first set up in countries where complementors could improve platform appeal to facilitate platform appeal and subsequently, entry in multiple countries. Stallkamp and Schotter (2021) explore another angle by distinguishing platforms with cross-country network externalities (i.e., the capability to create value by leveraging complementor contributions from current market(s) to a new country) and within-country network externalities (i.e., need for local complementors to create value in local markets). The authors posit that platforms with high within-country network externalities may enter foreign countries through joint ventures with or acquisition of a local platform.

Some researchers also draw attention to institutional challenges underlying managed ecosystem entry modes. Marano, Tallman, and Teegen (2020) note that asset-light and ‘permissionless’ entry of sharing economy platforms could later raise legitimacy challenges as their digital entry may not fulfil all legal requirements usually imposed on traditional incumbents. Kumar, Nim, and Agarwal (2020) show that regulations, infrastructure, and culture can influence adoption decisions of both retailers and users of mobile payment platforms. In particular, their study suggests that mobile payment platforms may successfully enter developing countries if they address institutional voids. Some scholars also take the perspective of complementors, suggesting that platforms entering foreign countries with institutional and infrastructure weaknesses may also help their complementors circumvent institutional voids to access the new market (Bei & Gielens, 2020; Sheth, 2020).

We summarize the main research insights on all four non-traditional entry modes in Table 1.

**Table 1:** A synthesis of the non-traditional entry mode literature*

		Capital access	Innovation outposts	Virtual presence	Managed ecosystems
Definition		Exploring foreign capital with little local embeddedness either through foreign debt or capital markets or by attracting foreign investors to home country	Embedding in foreign countries to establish informal networks with foreign firms for exploring new knowledge and resources	Exploiting technologies and capabilities in foreign countries through customer/user acquisition with low levels of local embeddedness	Exploiting multi-sided platform-based technology by embedding in foreign countries to create value through complementor contributions
Antecedents	Firm motivations	Gaining knowledge, managerial assistance, and reputation Insufficient capital availability at home	Accessing knowledge and resources Risk mitigation for future foreign entry	Overcome lack of resources and risks of foreign expansions	Enhancing platform value through complementor contributions
	Firm and country characteristics	Firm growth potential and relations with domestic investors Higher institutional quality at home country International experience and status of interested investors	Firm knowledge and absorptive capacity Network structure, knowledge overlap and willingness of network partners	Advances in digital technologies and emergence of global standards Firm technological and marketing competence, international experience, and client/user relationships	Firm-specific advantage, flexibility/generativity, and cross-country user networks, particularly in high clout countries Target country complementors offering prospects to improve platform appeal for multiple countries
Implications and consequences		Improved performance and higher engagement in foreign countries Coordination issues with foreign investors	Shift from formal information exchanges and weak ties to social exchanges and strong ties Higher structural and relational embeddedness	Diffusion-based user adoption Traditional entry after success in user acquisition	Overcome LOO to meet diverse user needs through complementor contributions Possible legitimacy challenges due to digital entry

## FRAMEWORK

Based on these insights, we develop theory to explain the use of these non-traditional entry modes. We recast international entry as a continuum in which firms attempt to exploit and/or explore for resources in foreign markets, instead of a firm’s boundary decision. We also suggest that embeddedness differs from location boundedness which describes the geographic fungibility of the core assets to be exploited (Verbeke & Hutzschenreuter, 2020). From an international strategy point of view, firms engaging in non-traditional entry are more concerned with how to achieve the required level of embeddedness in foreign locations to successfully explore for and/or exploit resources. A further distinguishing characteristic of our theory is that we do not view the exploitation-exploration continuum or embeddedness as determinants of firms’ entry mode selection decisions; they are instead conceptual dimensions by which we make sense of and categorize non-traditional entry modes, in much the same way as Williamson (1985) describes categories of organizational structures by attributes of incentive intensity and administrative authority.

Our conceptualization draws on two theoretical ideas that tend to link these categories of modes together. First is the notion that some entries are used primarily to exploit existing firm-specific resources while other entries are primarily used to identify and capture knowledge or other resources. These concepts originate from March’s (1991) seminal work regarding “exploration of new possibilities” and “exploitation of old certainties” are indicative of fundamental differences in firm behavior and strategy. In his seminal paper, March (1991: 71) refers to exploitation as “refinement, choice, production, efficiency, selection, implementation and execution,” contrasting it with exploration, which involves “search, variation, risk-taking, experimentation, play, flexibility, discovery, and innovation”. Levinthal and March (1993: 105) contend that exploration involves “a pursuit of new knowledge,” whereas exploitation involves “the use and development of things already known”.

This classic bifurcation has inspired much management research across a range of research fields, including innovation, organizational learning and organizational design, to name just a few (Lavie, Stettner, & Tushman, 2010). Researchers maintain that limited resource availability often compels firms to favor one of these activities over the other. Firms make conscious choices to allocate resources primarily to the refinement of existing technologies and the leveraging of existing competences or to the search for new resources and capabilities (Lavie et al., 2010). Meanwhile, researchers also stress that the concepts of exploitation-exploration encapsulate a continuum in which firms try to balance both activities in a particular situation (Gibson & Birkinshaw, 2004). The view among IB scholars is that firms can undertake exploitation or exploration activities jointly(Luo & Tung, 2018). MNEs’ foreign investment has long been viewed as a recombination of “extant reservoir of non-location bound FSAs being bundled with locally accessed, complementary resources and newly developed, location-bound FSAs” (Verbeke, Coeurderoy, & Matt, 2018: 1104), implying the concurrent importance of both exploitation and exploration.

The second theory that can be helpful in explaining non-traditional entry modes is the perspective of local embeddedness. Drawing on economic geography research, we define embeddedness by the extent to which the firm is anchored in a particular space to be integrated into, or generate, local networks of economic and social relations (Hess, 2004). Seminal research maintains that firms’ activities and outcomes are embedded in, and shaped by, their interorganizational relationships (Granovetter, 1985; Uzzi, 1996). Embeddedness arises as firms rely on intense interactions and close relationships with external stakeholders (such as suppliers, customers, and complementors) in obtaining the use of critical resources (Andersson et al., 2002; Nell & Ambos, 2013). During those exchanges and interactions, firms also develop relational codes and mutual commitments with stakeholders (Gulati & Gargiulo, 1999; Ring & van de Ven, 1994), establishing socioemotional affiliations that can shape network members’ social identities and direct their activities (Rao, Davis, & Ward, 2000). A key distinguishing feature of international business is the interaction across multiple contexts. Firms must adapt their organization and governance (e.g., entry mode choice) to the contextual differences that influence transaction and coordination costs as well as their resource advantages (Brouthers, et al., 2008). The local context has become an umbrella term referring to the institutional framework as well as the resource base that the MNE can access. Local embeddedness is critical as it facilitates such access and is a necessary condition for creating competence in a given context (Meyer, Mudambi, & Narula, 2011). At the subsidiary level, there also seems a correlation between the degree of local embeddedness and the ability to source new resources (Figueiredo, 2011).

The conventional view of internalization has drawn IB researchers’ attention to understanding the melding processes by which the MNE recombines existing resources with the new resources acquired in the target country (Verbeke, 2009). Such melding processes assume that various interlinked activities need to co-locate (in the host country) to create value. Similarly, research on MNE subsidiaries is also built on the premise that firms “must be ‘externally embedded’ within each local context” (Meyer et al., 2011: 236), emphasizing the need of embeddedness for knowledge creation and local adaptation (Tallman & Chacar, 2011). Meanwhile, much less is discussed as to whether and how the degree of firms’ interdependencies on local contexts may vary. We argue that while embeddedness may be endogenous and reflects the degree of a firm’s commitment to a particular location, there are also exogenous factors determining the degree of embeddedness such that different activities of resource exploitation or exploration may be by their nature more or less locally embedded. This intrinsic degree of local embeddedness further helps us classify non-traditional entry modes.

### Categorizing Non-traditional Entry Modes

We suggest that the first non-traditional entry mode, capital access, reflects a high level of exploration as it indicates firms seeking and obtaining new sources of capital in foreign countries. Such exploration appears to involve relatively low local embeddedness; firms may leverage the ease of global financial flows to obtain financial resources that requires little embeddedness in local networks of economic agents such as suppliers and customers. Exploitation, on the contrary, plays a limited role since the purpose of this type of entry is not to exploit ownership advantages to generate revenues but simply to help the firm capture financial resources needed within the organization.

The second non-traditional entry mode, innovation outposts, do not require significant foreign equity investments to obtain control of a specific technology. Instead, innovation outpost entry modes provide a continuous stream of knowledge which is dependent on the firm’s capacity to sufficiently embed itself into local networks of economic and social relations where strategic resources and new knowledge are situated (Vahlne & Johanson, 2017). Thus, these entry modes require firms to pursue a high embeddedness strategy at the same time as focusing on exploration of potential new knowledge that can be used throughout the firm to improve its competitive position, reduce the impact of potential new competitors, and offer opportunities to stay at the forefront of its industry.

The third non-traditional entry mode, virtual presence, highlights how digitalization has enabled firms to exploit digital channels in creating and delivering value to foreign customers/users. This entry mode emphasizes firms’ abilities to exploit firm-specific advantages and capabilities through digital technologies by remotely acquiring customers/users in foreign countries with little or no local embeddedness. It provides the opportunity for firms to exploit their advantages in multiple foreign markets with fairly low capital investments in any of these markets. Indeed, virtual presence entry mode provides the illusion of being locally embedded, especially when firms adopt local language and customs on the websites or are complementors on local multi-sided platforms, but in fact require little actual embeddedness in the local market. Hence, firms like Google (search engines), mobile app providers like Cheetah Mobile, or e-commerce retailers like BooHoo and SHEIN can be based in one country while providing their service/products to customers in other countries, through digital technologies, without having to embed in the local market.

Finally, managed ecosystem entry modes tend to involve a higher level of embeddedness as these specific platforms rely on complementors in each country for value creation and often have to engage extensively with local stakeholders in overcoming legal and other governmental barriers (Uzunca, Rigtering, & Ozcan, 2018). Resource exploitation would be untenable without the contribution of a network of complementors and acceptance in the local institutional context in which the platforms must be embedded. Thus, managed ecosystems provide a unique method of international entry facilitated by new digital technologies enabling firms to exploit existing resources in foreign markets through cooperation with local complementors and governments.

**Figure 2:**
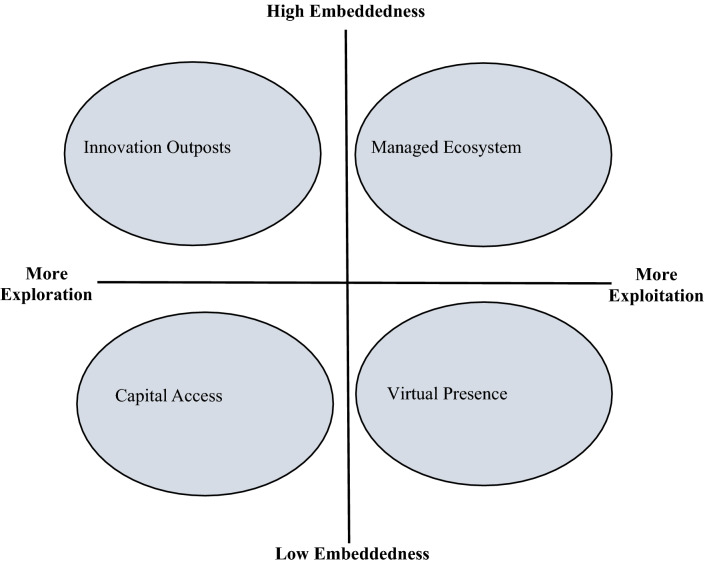
Mapping non-traditional entry modes.

To present our categorization of these non-traditional entry modes, we plot these four modes to the exploration, exploitation, and embeddedness (EEE) framework in Figure 2. While none of the mode categories is strictly limited to only exploration, exploitation, or low/high level of embeddedness, the relative positions of entry modes categories along the two continuums in Figure 2 reflect a higher orientation toward exploration or exploitation as well as a comparatively higher or lower need for local embeddedness.

In summary, what seems to have remained underplayed in the IB literature is the fact that some value-adding activities in foreign markets are inherently oriented toward exploitation “to ensure its current viability” and others toward exploration “to ensure its future viability” (Levinthal & March, 1993: 105). This has resulted in an under-appreciation of the distinction between exploitation and exploration in international entry research, a field predominantly inspired by the logic of transaction cost minimization. Despite the established view that firms increasingly perform foreign entries to gain access to resources and capabilities that can augment their core competencies (Dunning, 2000), only recently have entry mode scholars like Meyer, Wright, et al. (2009) reconceptualized traditional modes along the two dimensions of exploitation and exploration. Hence, we identify one dimension of a mode by its orientation toward the exploitation of existing resources and/or the exploration of new resources in foreign markets. We suggest these non-traditional entries represent a continuum of responses as firms might focus primarily on one activity while undertaking the other activity to a lesser/greater extent.

Furthermore, in some cases firms can enter a market without necessarily embedding in the local community while in other cases, as discussed by Johanson and Vahlne (2009) and others, entry requires significant embeddedness into local networks. The FDI-centric view dominating the IB literature has led researchers to focus on the combination of firm-specific assets with location-specific assets (Dunning, 1988; Jones, 2005). MNE subsidiaries are assumed to be a vehicle for internalizing location-specific assets when the external factor market fails (Hennart, 2009). Whether such resources can be traded on the market or are organizationally embedded in local firms determines how MNEs enter the market (Meyer, Estrin, et al., 2009). In a sense, ‘embeddedness’ shares some conceptual underpinning with what Hennart (2009) refers to as ‘asset bundling’, which views foreign market entry as the bundling of MNEs’ extant core assets with locally situated resources, whether or not through equity investment. Nonetheless, for non-traditional entry modes, embeddedness characterizes the need for firms to integrate themselves in local networks and engage with local stakeholders in order to undertake the task at hand (i.e., exploit internal resources or explore external resources). This need is, to some extent, assumed in the bundling perspective, which goes on to consider how to organize a transaction with those stakeholders in accessing complementary assets to be used locally. By contrast, we submit that the degree of embeddedness is not determined by the transactional characteristics of the assets being bundled; nor is embeddedness reliant on asset ownership.

### Non-traditional versus Traditional Entry Modes

We note that the EEE framework represents a rich integration of relevant theoretical frameworks, which has helped us categorize non-traditional entry modes on meaningful dimensions as well as provide the flexibility of incorporating new entry modes that research may identify in the future. To further demonstrate the distinctiveness of the non-traditional entry modes discussed above, we compare them with traditional entry modes in Table 2 based on the EEE framework as well as the familiar dimensions of cost and control (Anderson & Gatignon, 1986). The table reveals important distinctions among different entry modes as we show that each entry mode represents a different combination of exploration, exploitation, embeddedness, cost, and control. One key insight from this table is that our non-traditional entry modes offer novel configurations of exploration and exploitation opportunities with greater control but relatively lower costs, which were not available in entry modes that traditional mode research has focused on. For instance, virtual presence offers a similar level of exploitation opportunities as licensing or franchising, but grants greater control to the firm over customer interface and relationships. It also differs from service exporting given its focus on how the firm acquires customer/users and delivers value to customers/users, instead of how the product/service would be provided (e.g., via a distributor or sales subsidiary). Similarly, managed ecosystems and innovation outposts allow for exploitation and exploration opportunities respectively along with greater control like wholly owned subsidiaries, but at substantially lower costs. Finally, similar to the traditional modes of contracting, capital access modes do not rely on equity investment and have limited reference to control, thereby constituting a low-cost, low control mode of entry. Yet it differs significantly from contracting in that capital access facilitates a high degree of exploration, including the exposure to foreign ownership related resources such as managerial expertise, corporate governance practices, and reputation.

**Table 2:** How traditional and non-traditional entry modes differ

	Cost+	Control+	Exploration*	Exploitation*	Embeddedness
*Traditional entry modes*					
Wholly owned	H	H	H	H	H
Joint venture	M	H	M	M	M
Licensing	L	M	L	H	L
Franchising	L	M	L	H	L
Contracting	L	L	L	L	L
Exporting					
-Export from home	L	H	L	H	L
-Agent or distributor	M	M	M	M	M
-Joint venture	M	M	M	M	M
-Sales subsidiary	H	H	H	H	H
*Non-traditional entry modes*					
Virtual presence	M	H	L	H	L
Managed ecosystem	M	H	L	H	H
Innovation outposts	L	H	H	L	H
Capital access	L	L	H	L	L

H: High; M: Medium; L: Low.

+Anderson, E., & Gatignon, H. (1986). Modes of foreign entry: A transaction cost analysis and propositions. Journal of International Business Studies, 17(3), 1–26.

*Meyer, K. E., Wright, M., & Pruthi, S. (2009b). Managing knowledge in foreign entry strategies: a resource-based analysis. Strategic Management Journal, 30(5), 557–574.

Hence, we suggest that these new mode categories help us capture the unique aspects of non-traditional entry modes which are becoming more popular due to changes in technology and global institutions. With this new categorization, researchers should find it easier to identify, document, and discuss these less intensively explored entry modes.

## DISCUSSION

Our synthesis of extant research suggests that on the backdrop of dramatic changes in digital technologies and institutional frameworks, there is a growing research interest in non-traditional entry modes firms employ for foreign expansion. To guide future research in this direction, we synthesize currently disparate literatures on non-traditional entry modes to develop a theoretical ‘EEE’ framework based on March’s (1991) concept of exploitation/exploration and the notion of local embeddedness. This framework acknowledges that non-traditional forms of foreign entry may be driven by new considerations in addition to the transactional issues of control and cost. Instead, these non-traditional entry modes revolve around local embeddedness associated with a firm’s ability to exploit its current firm-specific advantages or explore additional advantages through external partners – investors, network partners, complementors and customers/users – in foreign countries. Our theoretical framework, based on a fast-emerging set of studies, is only the beginning of what promises to be a renaissance in entry mode research.

In this section, we discuss specific research directions for building on our EEE framework to advance the field of entry mode research. In particular, we highlight opportunities for expanding the EEE framework to shed light on firm choices of non-traditional entry modes as well as the need for deepening research on each of these entry mode types. Next, we draw attention to the choice firms make across non-traditional entry modes and between non-traditional and traditional entry modes. We point to new avenues for advancing the entry mode literature that not only incorporate new sets of mode choices but also configurations and constellation of traditional and non-traditional entry modes.

### Deepening Research on Non-traditional Entry Modes

While there is an emerging literature on non-traditional entry modes, further research is needed to better delineate the driving forces and barriers to explain why firms choose these entry modes and what governance arrangements are required for these unique relationships with investors, network partners, complementors and customers/users. Below, we explain how our theoretical framework can guide future research in these directions.

#### Driving forces

We propose a framework of non-traditional entry modes based on the need for embeddedness and the strategic choice of exploration or exploitation. Although the strategic trade-off between exploration and exploitation is widely discussed in the strategy literature (Benner & Tushman, 2015; Uotila, Maula, Keil, & Zahra, 2009) and our synthesis of the literature reveals entry modes consistent with exploration or exploitation activities, there is still little understanding about the influence of firm motivations on non-traditional entry mode choices. A fundamental question in this regard is the nature of knowledge, resources, and capabilities that firms prefer to explore or exploit through non-traditional entry modes, by engaging with network partners, investors, or complementors, instead of employing traditional modes. Are these choices driven at least in part by the location-bound nature of firm-specific advantages? To what extent are firm-specific as well as location-specific advantages required for exploration or exploitation through non-traditional entry modes? Researchers may also move beyond the nature of knowledge – i.e., tacit nature of knowledge, complexity, and codifiability – held by firms (Kogut & Zander, 1993) to consider the tacit or codified nature of knowledge held by external partners. The latter may determine firm motivation as well as capacity to explore/exploit and the need for local embeddedness, and ultimately the mode choice.

A deeper question is related to the combined motive of both exploitation and exploration and its impact on entry mode choices. Can non-traditional entry modes support simultaneous exploration and exploitation in foreign entries? Will it require firms to pursue new entry modes not yet covered in the literature and would it require the simultaneous pursuit of multiple different entry modes in a market? Future research needs to examine the impact of such trade-offs between and within these different strategies on entry mode choices.

Researchers also need to elaborate on how local embeddedness will drive non-traditional entry mode choices as we know little about how firms determine the level of embeddedness needed in a specific market. In particular, researchers may explore the interaction with local institutions and the impact of different forms of distance. Are firm decisions about embeddedness, and corresponding non-traditional entry modes, influenced by local institutions (such as changes in laws and regulations) or by the actions of consumers (buyers/users) or network partners? Another critical question is related to the requirements for achieving and managing local embeddedness in foreign markets, which may determine firms’ decisions to pursue non-traditional entry modes. Once a firm decides on the right level of embeddedness in a foreign country, how is this task achieved in an efficient and effective manner? Do firms need large financial investments to enhance their embeddedness or can the cultivation of internal or external networks earn firms legitimacy and facilitate their embeddedness? What alternatives do firms have to establish, increase, or decrease the level of embeddedness in a foreign market?

#### Barriers to non-traditional entry modes

In addition to firm motivations that may drive non-traditional entry mode choices, it is equally imperative to consider the challenges that firms pursuing these entry modes may face. In particular, recognizing non-traditional entry modes employed by modern businesses extends our understanding of the challenge of overcoming LOO. Traditional entry modes usually focus on LOF, discussing how firms overcome institutional differences through mode choices (Hennart & Larimo, 1998; Kogut & Singh, 1988). Firms employing non-traditional entry modes, on the contrary, face various forms of LOO regarding investors, network partners, complementors, legal entities, and customers/users in foreign countries. Yet theoretically, there is little understanding about the challenges imposed by LOO and the trade-offs firms make to move from outsiders to insiders as they transition from one foreign market to another or become embedded in a particular market. With the paradigm shift from LOF to LOO, researchers should focus more on the impact of LOO on firm capabilities for identifying foreign partners and cultivating the right relationships required for non-traditional entry modes. Further, there is an increased need to note different sources of the LOO and measures through which MNEs can overcome the LOO. What theoretical perspectives, such as the OLI framework, resource-based view, or network theories, can help advance our understanding of LOO? Are there specific mechanisms that can help firms achieve insider status in an effective and efficient way?

Meanwhile, we acknowledge that many barriers to traditional foreign entry may still hold for non-traditional entry modes but current research lacks a systematic assessment of the relevance of traditional barriers for non-traditional entry modes. Future research needs to determine the extent to which institutional distance as well as institutional profiles of target countries impede or facilitate non-traditional entry modes. Even regarding digital entries, there is an increased recognition of the role of regulatory differences across countries (Ghosh, 2018; Uzunca et al., 2018), which researchers need to take into account. Clearly, considering both traditional and novel barriers is necessary to provide a comprehensive view of the challenges firms pursing non-traditional entry modes face.

#### Governance

A unique aspect of non-traditional entry modes is the emergence of new channels to access resources and capabilities which may not necessarily require contractual arrangements pertaining to knowledge and resource exchanges. While traditional MNEs are products of internalization and control, modern MNEs may benefit to a greater degree from externalization, including network externalities, the separation between foreign investors and managers, and customer/user acquisition through digital channels. Such a change in focus may also require changes in governance arrangements as control structures may need to be replaced with network management and relationship building, which requires a new lens to entry mode governance research.

In particular, as formal contracts may play a less important role in non-traditional entry modes, usual tools such as due diligence may be of less relevance; firms may not expect their partners, complementors, and users to reveal information beforehand. Hence, we need a renewed understanding of ex-ante mechanisms for uncovering the knowledge and resources held by investors, network partners, complementors, and customers/users in the absence of formal tools such as due diligence or voluntary disclosures. Mainly, researchers may shift focus to the informal channels firms can rely on to ascertain the nature and utility of knowledge and resources they want to explore or exploit through non-traditional entry modes.

Equally important are ex-post governance of semi-formal and informal relationships to ensure adequate exploration or exploitation and local embeddedness. An important consideration for firms pursuing non-traditional entry modes is to mitigate opportunistic behaviors by network partners, platforms, and complementors (Deng et al., 2021). This may not require greater completeness in contracting but management of deeper, trust-based relationships. Researchers need to evaluate how firms manage the trade-off between knowledge exchange opportunities from non-traditional entry modes and the risks inherent in opening their information, technology, or platforms to external partners? Under which conditions can such governance challenges induce firms to avoid non-traditional entry modes and pursue more traditional entry modes?

The question of governance is also critical in view of the corollaries between non-traditional entry modes and the emergence of new organizational designs that may either flatten the firm or confer more centralized control to headquarters through greater transparency of overseas operations. Indeed, non-traditional entry modes provide a lens to explore the tension between centripetal and centrifugal forces of digitalization (Autio et al., 2021). On the one hand, digitalization acts as a centrifugal force as non-traditional entry modes facilitate firm internationalization in diverse countries. On the other hand, advanced digital tools to centrally coordinate or even micro-manage ever-expanding global operations may reduce the relevance of locally embedded resources, management teams, and subsidiaries. In view of new technologies such as block chain, virtual office, and AI capabilities, future research may investigate the impact of technology on modes of governing international subsidiaries and external partners to improve the efficiency and effectiveness of existing governance mechanisms. At the same time, the information overload, emanating from the availability of big data about internal and external partners and customers/users across the globe, may undermine managerial capacity and may require novel approaches to international corporate governance (Clough & Wu, 2022; Raisch & Krakowski, 2021; Verbeke & Hutzschenreuter, 2020). Building on these ideas, future research could explore issues related to corporate governance in these new MNEs and between headquarters and networks of partners, investors, and complementors established through these non-traditional entry modes.

### Research Questions Specific to Non-traditional Entry Modes

In addition to future research questions common to all non-traditional entry modes, we also dive deeper to highlight questions specific to each non-traditional entry mode category.

#### Capital access

While prior studies have explored various factors that drive VC investment and success of foreign IPOs, little consideration is given to the codified or tacit knowledge of firms as well as investors. For example, to what extent does tacit knowledge of VCs or investors in a country motivate firms to access capital markets without entering country product markets, especially when the firm itself possesses highly codified knowledge that may not enhance its advantage or is difficult to protect in foreign product markets? Does the free flow of financial capital also facilitate the flow of knowledge or intellectual capital across countries? Equally important is research on potential risks arising from differences in compliance standards or signing VAM (Valuation Adjustment Mechanism). Another important research area is related to the roles of individual investors/brokers in the process. Researchers may explore implications of such entries for stakeholders – individual and/or institutional investors, entrepreneurs, policy makers, and overall communities – in both home countries of firms and host countries providing capital.

It is also crucial to look at mode choices within this non-traditional entry mode (Filatotchev et al., 2016); firms choose between debt and equity and within each of these categories, among a multiplicity of alternatives such as foreign IPO, VC funding, cross-listings, foreign bonds, Eurobonds, bank credit, and convertible bonds. Researchers evaluating such choices may not only draw from transaction cost economics and agency theory that are usually considered in finance research, but also employ novel theoretical lenses from institutional theory, resource-based view, or the knowledge-based view (Purkayastha & Kumar, 2021). There may be important considerations related to knowledge exchanges, coordination issues due to distance, LOF or dynamic LOF considerations, firm experience, and even micro-foundations such as managerial preferences, which do not naturally fall in the finance domain but are critical to advance an IB understanding of this entry mode choice. Prior research also identifies a distinct path through which platform owners utilize VC investment to cultivate a managed ecosystem around them (Tong, Guo, & Chen, 2021), suggesting an interplay between capital access and other non-traditional modes.

#### Innovation outposts

Innovation outposts bear a close relation with the literature on the ‘global factory’ (Buckley & Ghauri, 2004) and ‘global value chains’ (Kano et al., 2020). However, given the fast-changing nature of the global business landscape such as the rise of nationalistic sentiments, a trend toward de-globalization, and global pandemics like COVID-19, both scholars and practitioners need to pay attention to exogenous shocks in the global system, and particularly the implications of gray rhino (i.e., foreseeable catastrophes that spread across borders, see Wucker, 2016) or black swan (i.e., highly improbable events that have a major impact if they occur, see Taleb, 2007) events for not only formally organized global value chains but also informal and trust-based networks of global partners. How can MNEs develop more agile and resilient networks in overseas markets is a critical research question in view of such transitions. In addition to firm-level capabilities and strategies, researchers may also evaluate the role of foreign regulations and institutional environments in facilitating or impeding firms’ cultivation of such informal relationships as well as the sustainability of such networks. Overall, in addition to the focus of extant research on the benefits of embedding in a foreign host country to better learn and absorb knowledge, future research also needs to explore how institutional multiplicity in home and host countries will affect the degree of embeddedness in a host country as well as the potential risks of espionage and even expropriation.

Future research may also shed light on firm choice between traditional entry, through internalization and control, and innovation outposts. One question, for instance, is whether entry based on innovation outposts may be more beneficial when partners hold tacit knowledge that is hard to explore through contract-based relationship. Similarly, are traditional entry modes sufficient or necessarily more efficient when partners offer largely codified knowledge? Given that firms pursue valuable, rare, and inimitable resources, can firms gain a resource-based advantage through innovation outposts since the opportunity of establishing foreign networks may be widely available to their competitors as well? Is it necessary for firms to switch from innovation outposts to more traditional entry modes for attaining competitive advantage? There is clearly a vast opportunity of research on the relationship between innovation outposts and traditional entry modes.

#### Virtual presence

With the development of digital technologies, an increasing number of firms are now engaging directly with customers/users via digital channels, such as software-as-a-service (SaaS), platform-as-a-service (PaaS), or infrastructure-as-a-service (IaaS), to name a few. While current research focuses on the exploitation of technology or capabilities for revenue generation across borders (e.g., Monaghan et al., 2020; Shaheer & Li, 2020), more research is needed that focuses on the learning and value co-creation opportunities that firms can enjoy by acquiring customers/users in new countries. Such cross-pollination of knowledge and learning across borders may play a critical role in improving firm technologies and capabilities, and may serve as an important motivation for acquiring customers/users in foreign countries, beyond a simple market-seeking logic.

The literature also needs to acknowledge more nuances in the entry of virtual presence as many digital and professional service firms not only virtually transmit digital technologies or provide services from overseas offices but also establish complementary off-line facilities to enhance customer value in given regions. In fact, more traditional MNEs are also transitioning to digital business models, reducing their physical footprint and focusing on technology and digital channels to expand abroad. Little do we know about why and when firms combine both online and offline approaches in serving foreign markets.

Another important but largely neglected area of research is related to global entrepreneurship opportunities for individuals and users (Chandra & Coviello, 2010), who can now enter foreign countries to acquire clients and users through digital channels. Such foreign entries are not limited to only app developers or software engineers. There are an increased number of professionals acquiring foreign clients for consulting, education, and design services on digital platforms. Such opportunities lead to the emergence of ‘micromultinationals’ and individual entrepreneurs across countries and play a vital role for many economies. Still, current research sheds limited light on key success factors and challenges for such foreign entries by individuals and micro firms.

#### Managed ecosystems

With the development of digital technology, MNEs can manage their economic activities through single-sided or multi-sided platforms, which may influence their level of local embeddedness. We identify at least three types of platform firms. Single-sided platforms like clothing firms, or streaming services such as Netflix or Disney+ may require very low host country embeddedness since their products are non-location bound and can be seamlessly transmitted to any country. Hence, these organizations ted to rely on virtual presence entry modes for international expansion. Some multi-sided platforms (like eBay or Uber) require greater embeddedness in foreign markets as they rely on contributions by local complementors as well as customers/users who value the offerings of those complementors. However, local embeddedness may vary based on the location-boundedness of complementors; complementors on platforms like eBay are not always location bound, enabling the platform to penetrate some countries with fewer local complementors. On the other hand, complementors of sharing economy platforms like Uber have high location boundness, rendering local embeddedness critical for successful penetration. Other multi-sided platforms may require very high levels of foreign market embeddedness since all three legs of the business model (i.e., suppliers, delivery services, and consumers) are embedded in the local market and are location-bound. Thus, future research needs to distinguish platforms based on their characteristics to better understand the deeper nuances within and between virtual presence and managed ecosystem entry modes. Indeed, even traditional MNEs are employing platforms like crowdsourcing or internet of things to access knowledge and resources in new and existing countries, which IB research should explore.

### Integrating Non-traditional and Traditional Entry Modes: Choices, Constellations, and Configurations

While current research largely focuses on four major non-traditional entry mode categories that correspond to our theoretical framework, yet our framework is not confined to discrete mode types but builds on two continuums of exploration/exploitation and embeddedness. Hence, there really exist a much larger number of entry modes within these categories than we currently depict as firms can pursue several entry modes within the continuum between exploration and exploitation as well as between high and low embeddedness. With recognition of this variety of entry modes, we suggest that scholars can go beyond treating foreign entry as a discrete choice to explore deeper questions about how MNEs orchestrate their entries by choosing a combination of entry modes out of a large pool of traditional and non-traditional entry modes. Below, we discuss how we can cross-fertilize research on non-traditional and traditional entry modes to discuss not only mode choices but also configurations and constellations through which firms embed in foreign countries for resource exploration, exploitation, or a combination of both.

#### Entry modes choices

One main objective of our paper is to acknowledge non-traditional ways for entering foreign countries, which could provide researchers with a more complete set of entry mode choices available to firms, in addition to traditional greenfield investment, acquisitions, joint ventures and wholly owned subsidiaries. This is a critical development in entry mode research as firms often make mode choices that are between traditional and non-traditional entry modes. For instance, digital platforms that are often considered non-location bound are pursuing more integrated modes by investing in physical infrastructure (Stallkamp & Schotter, 2021). Similarly, more traditional firms can enter foreign markets not only through traditional entry modes but also by initiating platforms and orchestrating a surrounding ecosystem (Nambisan et al., 2019).

We suggest that researchers go beyond conceptualizing entry modes as binary choices to acknowledge the processes and mechanisms of entering foreign markets, which require firms to make several strategic choices during their foreign entry into a country. Unlike traditional market entries where making physical investments or entering into a contractual arrangement can be genuinely considered the entry point, non-traditional entry modes may not entail such clear-cut events. Capital access may be an exception where success in obtaining foreign funds can mark an entry but other modes tend to unfold over a process of entry without discrete entry points. For example, digital firms or platforms may gain an initial foothold before penetration, which may enable them to iterate and improve their offerings to complete the penetration process (Chen, Wang, et al., 2021; Chen, Zhang, et al., 2021; Chen, Zhang, Li, & Turner, 2021b). The drivers, strategies and trade-offs that lead to an initial foothold and penetration can be very different from each other and may require separate research streams employing different theoretical perspectives. Similarly, establishing innovation outposts is a process that can possibly be dissected into multiple components for identifying key success factors, challenges, and strategic choices within each phase. Taking a process perspective of foreign entry and evaluating choices over the entire process can open a new venue for extensive research on entry modes.

#### Entry mode configurations

Entry mode configuration refers to the sequence of entry modes adopted in a given country. A broader view of entry modes may unveil more nuances as to how firms evolve their entry mode choices and enhance or reduce their commitment in different countries. For instance, it is possible that firms may initially enter a foreign market by establishing innovation outposts and virtual presence and later choose the same or different markets for traditional entry. An important example is Dropbox, which entered many European markets through virtual presence channels, but later installed hardware infrastructure in some countries and established sales and technical support offices in others (Shaheer, Woo, Stallkamp, Li, & Chen, 2021). Hence, we encourage research on patterns of mode choices in early and late stages of the internationalization process, which could provide a more fine-grained understanding of firm entry mode sequence. Such an inquiry may also reveal how firms configure different entry modes to effectively operate in a foreign market to co-create value with local stakeholders, which may further improve our understanding of entry modes as modes of operation.

Researchers may explore if such relationships with external partners can influence subsequent entry mode choices in the same country through different processes such as mimetic, coercive, osmotic, and apprentice (see Borgatti & Halgin, 2011 for details). To what extent do firms learn from the experiences of their partners and at times, follow their partners, which reflects a mimetic or apprentice process of influence? Firms may also expect support from their partners in pursuing certain entry modes. For example, firms can receive support from VCs for establishing a joint venture with another foreign firm, or obtain greater appeal from existing complementors by acquisition of another platform, both of which seem to indicate a coercive process. An important example is ByteDance which entered the US market through an acquisition of Musical.ly, partly to increase its appeal and influence among its Asian complementors who would value and benefit from the Musical.ly network of American users and complementors (Wang, Shaheer, Li, Chen, & Yi, 2019). Firms can also make entry mode decisions to maintain or cultivate their network positions as a bridge among partners or to strengthen their status or reputation among network members. Hence, we suggest considering how firms’ prior entries through non-traditional entry modes and resulting network characteristics in which firms are embedded as well as the network positions firms hold, can shed new light on the pursuit of non-traditional or traditional entry modes.

A focus on entry mode configurations may also shed new light on why and how MNEs exit or reduce commitment from a given foreign market. In view of non-traditional entry modes, the closing or selling-off of physical facilities may not necessarily indicate an exit from a market as firms may maintain their presence through a digital platform or remote office. In this regard, an exit does not necessarily indicate a firm’s failure in a given market as it may simply reconfigure mode choices within the market to more efficiently serve its stakeholders. Future research needs to explore these ‘configurational dynamics’ and not assume firms exit and re-enter markets when in reality they are reconfiguring existing modes to better serve a market.

#### Entry mode constellations

Traditional entry mode research tends to emphasize control and commitment, seeking an ‘ideal’ or most efficient governance mode. But we acknowledge firms investing overseas can have multiple modes coexisting to better dovetail with the synergy and value in different foreign markets. By highlighting non-traditional modes, we hope future research will keep exploring how MNEs can manage the constellation of different modes in foreign markets to create value. An important example is Facebook which enters some countries via virtual presence modes while establishing local offices and digital infrastructure in others. Netflix, Zoom and Dropbox, which mainly focus on virtual presence modes of entry, either own or rent data storage hubs from third parties/platform providers like Amazon AWS to store complementors data locally, thus requiring more traditional modes. Investigating what factors drive the different combinations of entry modes in international markets provides important research opportunities.

Similarly, many traditional firms are entering new countries or initiating new entry modes in current markets based on virtual presence or managed ecosystems, which allows these firms to conduct activities ranging from trading and marketing to crowdsourcing and user co-creation, all lying across different levels of exploration or exploitation and local embeddedness. Indeed, associating the primary entry modes with certain industries may substantially limit research potential of uncovering how MNEs enter and operate in foreign markets. There are numerous possible constellations of entry modes firms adopt as they choose across different levels of embeddedness, taking different positions in capability exploration and exploitation, or orchestrate different types of business operations in different countries based on their changing needs, motivations, institutional environments, and market characteristics. It might be fruitful for future research to further examine the factors leading to different constellations or combinations of entry modes, and their practical implications.

## CONCLUSION

Technological advancements and improved global connectivity are providing businesses novel ways to enter foreign countries. Instead of ownership and control, the non-traditional modes we highlight in our study largely focus on local embeddedness for exploration and exploitation of knowledge and resources. Recent IB and management research also acknowledges these non-traditional modes, though the literature on non-traditional entry modes remains largely fragmented and disconnected from the broader entry mode literature. Because of this, current research is missing the vital opportunity to inform entry mode research and create opportunities for the cross-fertilization between non-traditional and traditional entry mode literatures. We take an initial step toward incorporating non-traditional entry modes into the mainstream entry mode literature by consolidating developments in non-traditional mode research, highlighting areas for advancing this research, and integrating traditional and non-traditional entry mode research streams. While Hennart and Slangen (2015) recognize the potential of bringing further depth to the entry mode literature, introducing non-traditional entry modes highlights the breadth of entry mode choices that need to be incorporated in current research. It is worth noting that technological and institutional foundations are in continuous flux. New technologies such as block chain, virtual office, and AI capabilities as well as socio-political transitions may lead to the emergence of further non-traditional modes that are yet not covered in the literature. Hence, researchers should be on continuous lookout for opportunities to enhance the breadth of entry mode literature. Only through more concrete efforts in understanding how firms choose across a much larger set of entry modes, can we develop new theory and advance current frameworks to improve our knowledge of international entry and remain relevant to international business practice.
